# Generation of Gross Chromosomal Rearrangements by a Single Engineered DNA Double Strand Break

**DOI:** 10.1038/srep43156

**Published:** 2017-02-22

**Authors:** Zhijun Qiu, Zhenhua Zhang, Anna Roschke, Tamas Varga, Peter D. Aplan

**Affiliations:** 1 Genetics Branch National Cancer Institute, National Institutes of Health, Bethesda, MD 20892, USA

## Abstract

Gross chromosomal rearrangements (GCRs), including translocations, inversions amplifications, and deletions, can be causal events leading to malignant transformation. GCRs are thought to be triggered by DNA double strand breaks (DSBs), which in turn can be spontaneous or induced by external agents (eg. cytotoxic chemotherapy, ionizing radiation). It has been shown that induction of DNA DSBs at two defined loci can produce stable balanced chromosomal translocations, however, a single engineered DNA DSB could not. Herein, we report that although a single engineered DNA DSB in H2AX “knockdown” cells did not generate GCRs, repair of a single engineered DNA DSB in fibroblasts that had ablated H2ax did produce clonal, stable GCRs, including balanced translocations and megabase-pair inversions. Upon correction of the H2ax deficiency, cells no longer generated GCRs following a single engineered DNA DSB. These findings demonstrate that clonal, stable GCRs can be produced by a single engineered DNA DSB in H2ax knockout cells, and that the production of these GCRs is ameliorated by H2ax expression.

Gross chromosomal rearrangements (GCRs), including chromosomal translocations, inversions, amplifications, and deletions are associated with most cases of cancer ((Mitelman Database of Chromosome Aberrations in Cancer, at http://cgap.nci.nih.gov/Chromosomes/Mitelman))^1^. In some cases, particularly those recurrent, non-random chromosomal translocations associated with hematopoietic malignancy, epidemiologic and experimental data have demonstrated that these GCRs are direct causes of malignant transformation^2^. Given that at least some GCRs are causal events for malignant transformation, it becomes important to understand the mechanisms that lead to these GCRs in order to understand the root causes of cancer, and develop novel therapeutic approaches to cancer. Because of the recurrent and non-random nature of chromosomal translocations associated with hematopoietic malignancies, it has been attractive to predict that these recurrent GCRs involve regions of the genome that are peculiarly “recombinogenic”, and likely to recombine specifically, i.e., BCR with ABL, as opposed to BCR with GAPDH^3^,^4^,^5^. However, it is also possible that BCR-GAPDH fusions are more common than BCR-ABL fusions, but that the BCR-GAPDH fusions do not clonally expand and lead to leukemic transformation, thus do not come to medical attention, and remain undetected in a pool of polyclonal hematopoietic cells. Therefore, in order to study “neutral” GCRs an assay that does not give the cells undergoing the GCR a selective advantage is required.

Although the cells of individuals with inherited DNA repair defects (Fanconi’s anemia, ataxia telangiectasia, Nijmegen’s breakage syndrome, Bloom’s syndrome) are predisposed to GCRs^6^,^7^, the majority of individuals with oncogenic chromosomal rearrangements have no known inherited predisposition. Based largely on the analysis of translocation breakpoint sequences and model systems, several mechanisms have been proposed to explain the recurrent translocations seen in leukemic cells^2^. These include (i) illegitimate VDJ or switch recombination^8^,^9^,^10^, (ii) homologous recombination mediated by repetitive sequences^11^,^12^, (iii) DNA topoisomerase II subunit exchange^13^,^14^, and iv) error prone non-homologous end-joining (NHEJ) repair of a DNA double strand break (DSB)^15^,^16^,^17^. These proposed mechanisms are not mutually exclusive and some recurrent translocations are derived from interaction of different mechanisms; for instance, a translocation breakpoint may show evidence of cleavage at a V(D)J signal sequence, and repair via a proposed error prone NHEJ pathway^18^. The most common mechanism implicated seems to be inappropriate repair of one or more DNA DSB by either the classic or error-prone, alternative NHEJ pathways.

In order to address questions such as which forms of GCR (amplification, deletion, inversion, translocation) are most common in the absence of oncogenic selection for specific GCRs, and whether defects in DNA DSB repair pathways would lead to increased frequency of GCRs, again in the absence of oncogenic selection for specific GCRs, a number of investigators have developed experimental systems in which a single DNA DSB can be introduced into a mammalian genome^19^,^20^. The recognition site for the meganuclease I-SceI, whose 18 bp recognition sequence does not normally occur in the mouse or human genomes, is introduced into mouse or human cells, and a single DNA DSB is produced by expression of the I-SceI enzyme. In theory, aberrant repair, involving a second, random DNA DSB could produce a large (megabase) deletion, translocation, or inversion. However, we were unable to detect any clonal, stable GCRs (GCRs that are stable and found in a clonal population of daughter cells), such as balanced chromosomal translocations or megabase chromosomal inversions, following the introduction of a single, I-SceI- engineered DNA DSB^21^,^22^. These findings were consistent with previous reports that the frequency of chromosomal translocations induced by a single DNA DSB in mouse embryonic stem (ES) cells was extraordinarily rare (<5 × 10^−8^)^23^. In this report, we modify the experimental approach to determine if clonal, stable GCRs can be produced in cells that are deficient for DNA repair components, such as NHEJ proteins or H2ax, a variant histone which becomes phosphorylated in response to DNA DSB and “coats” the DNA in the region of the DNA DSB^24^.

## Results

### Inhibition of NHEJ does not lead to frequent GCR following a single DNA DSB in human U937 cells

In an effort to generate GCRs *in vivo*, we previously generated a cell line, (designated F5) that had incorporated a single copy of the EF1aTK vector, which uses the EF1a promoter to drive expression of the herpes simplex thymidine kinase (TK) gene^21^. A recognition sequence for the I-SceI restriction enzyme was placed between the EF1a promoter and the TK cDNA, thus, separation of the EF1a promoter from the TK gene leads to lack of TK expression. The TK protein can phosphorylate the prodrug ganciclovir (GCV) producing the nucleoside analog GCV triphosphate, which causes DNA chain termination and subsequent apoptosis^25^. Therefore, a cell line with an intact EF1aTK vector produces the TK enzyme, and is sensitive to GCV, whereas a cell that has lost TK expression becomes resistant to GCV. Millions of cells can be quickly screened for GCV resistance, due to loss of TK expression. We transfected an I-SceI expression vector into the F5 cells, reasoning that a rare mis-repair of the cells would lead to a GCR characterized by separation of the EF1a promoter and the TK cDNA, loss of TK protein expression, resulting in GCV resistance (GCV^R^). Following transfection of an I-SceI expression vector, we isolated pure, single cell clones and characterized hundreds of GCV^R^ clones, however, none showed a GCR. Instead, all of the clones cleaved by I-SceI had become GCV^R^ due to deletion of portions of the EF1a promoter or TK cDNA, or to insertion of foreign DNA at the I-SceI cleavage site^21^,^22^.

**Figure 1:**
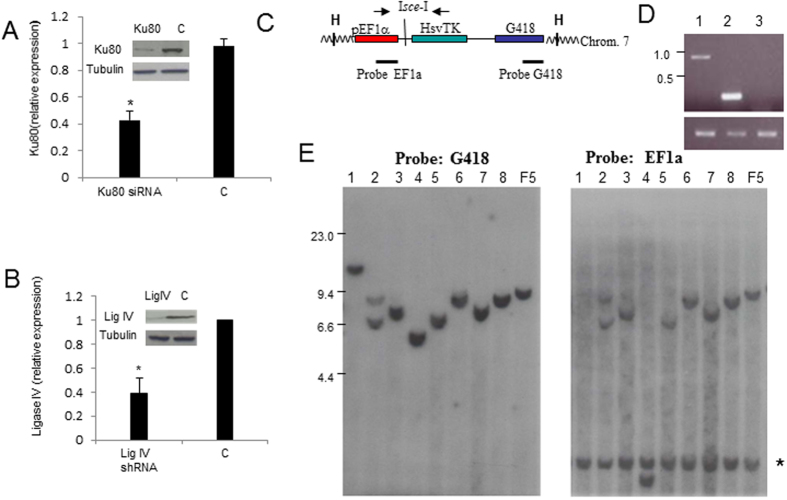
Knockdown of KU80 or LIG4 does not lead to GCR. (**A**) Knockdown of KU80 in F5 cells. Relative expression of KU80 mRNA in cells transfected with a KU80 siRNA or scrambled control (C), normalized to 18 S RNA. * indicates p < 0.01. Inset represents Western blot of KU80 protein; tubulin is used as a loading control. Blot cropped to improve clarity and conciseness. (**B**) Knockdown of LIG4 via shRNA. Control is MISSION^®^ pLKO.1-puro Non-Mammalian shRNA Control. LIG4 mRNA and protein were assessed as in panel A. Western blot cropped to improve clarity and conciseness.(**C)** Map of EF1a-TK cassette integrated on chromosome 7 in U937 cells. EF1a, HsTK, and G418 sequences are indicated; H indicates a Hind III site. There are no Hind III sites within the vector. The locations of primers and DNA fragments used as probes are shown. (**D**) Analysis of GCVR clones with PCR primers flanking the I-SceI site. WT fragment (not shown) is 404 bp. The clone in lane 1 (clone 1-1) has an insertion, the clone in lane 2 (clone 2-1) has a deletion, and the clone in lane 3 (clone 3-4) does not amplify, indicating that the primer binding sites have been deleted or separated. Amplification of SVCT (below) is used as a DNA quality control. Ethidium bromide stained gel cropped to improve clarity and conciseness. (**E)** Hybridization of Hind III restricted DNA from 8 clones that could not amplify DNA across the I-SceI site (such as clone 3–4 in panel D) to the EF1a or Neo probe. Since there are no Hind III sites within the EF1a-TK vector, the G418 and EF1A probes hybridize to an identical fragment in the F5 control lane. The clones in lanes 2, 3, 5, 6, 7, 8 hybridize to identical, non-wild-type fragments, indicating an interstitial deletion. The clone in lane 1 has deleted the exogenous EF1a fragment, and could represent a large interstitial deletion. The clone in lane 4 (designated KU4-27) has different, non-wildtype size fragments hybridizing to the G418 and EF1a probes, and could represent a GCR or large insertion, that contains at least one Hind III site. Size standard are in kb; * indicates the endogenous EF1a fragment.

To determine if inhibition of the “classical” NHEJ pathway would be permissive for GCRs in such a system, perhaps due to activation of an “error-prone”, alternative NHEJ^15^, we used RNAi to inhibit expression of KU80 or LIG4 in the F5 cells which harbored the EF1aTK vector. F5 cells were co-transfected with a KU80 siRNA pool and an I-SceI expression vector (pCBASce). After a 96 hour incubation period (to allow the I-SceI protein to be expressed, create a DNA DSB, mis-repair, and eliminate TK expression), individual GCV^R^ clones were obtained by limiting dilution in the presence of 50 uM GCV. Alternatively, LIG 4 production was inhibited by transduction with a lentiviral vector that expressed a short hairpin (sh) RNA directed against LIG4. As shown in Fig. 1A, KU80 transcripts and KU80 protein synthesis (assayed at 48 hours after transfection) were both reduced. Figure 1B shows a similar degree of inhibition for LIG4. A total of 189 clones were obtained after KU80 knockdown and GCV selection; these clones were assessed for evidence of GCRs using a combination of PCR and Southern blots, as previously described and outlined in Fig. 1C–E 21,22. The majority (124/189) of the single-cell clones recovered were false positive clones in which the I-SceI site remained intact and had become GCV^R^ by an alternate mechanism (Table 1). Sixty clones had small (defined as <2.3 kb) deletions encompassing the I-SceI site, and three clones had larger (>2.3 kb) deletions flanking the I-SceI site, indicating that the GCV resistance was due to cleavage at the I-SceI site followed by resection of DNA and re-ligation, leading to an interstitial deletion and loss of TK protein expression (Table 1).

Based on the Southern blot and PCR results, two clones were candidates for GCRs (see lane 4, Fig. 1E). We used inverse PCR to clone the junction sites for these candidates. One clone (designated KU4-27, lane 4 in Fig. 1E) had undergone a local inversion involving adjacent chromosome 7 sequences of 15,116 bp. A second clone (designated KU14) fused 706 nucleotides derived from chromosome 8 DNA (nucleotide 8:130542186-130542892) to TK sequences, and was regarded as a potential GCR. To determine whether this represented a GCR, as opposed to a templated sequence insertion (TSI)^26^ of chromosome 8 material, we used fluorescent *in situ* hybridization (FISH). A BAC probe 5 kb telomeric to the chromosome 8 fusion [8:130548708-130693512; (RP11-419K12)] was labeled with rhodamine and a chromosome 7 BAC probe that was 7 kb centromeric [7:112208320-112269438; (RP11-467K21)] to the integration site of the EF1aTK construct was labeled with FITC. The two probes were hybridized to metaphase spreads of the F5 parental cell line and the KU14 daughter clone (Supp Fig. 1). However, the signals did not co-localize in the KU14 clone, indicating that the KU14 cell line had not undergone a GCR. A control experiment in which the I-SceI expression vector only was transfected (no siRNA) yielded only interstitial deletions, similar to our previous observations^21^, with no evidence for GCV^R^ clones that had undergone a GCR (Table 1).

A clone which markedly inhibited LIG4 expression (Fig. 1B) was transfected with the I-SceI expression vector, and 130 GCVR clones were identified (Table 1). The vast majority of these clones were either false positive clones or interstitial deletions, however, one clone had a local inversion involving adjacent chromosome 7 sequences of 14,927 bp, and a second clone, designated 4-70-3-15, had a 35 Mb interstitial deletion that was verified by FISH (Supp Fig. 2). In sum, of 319 total clones obtained by inhibition of NHEJ and an engineered DNA DSB, 160 were false positive, and a single clone had undergone a GCR (35 Mb deletion).

### Inhibition of H2AX does not lead to frequent GCR in U937 cells

Given that inhibition of the classical NHEJ pathway did not lead to frequent GCR, we investigated the possibility that inhibition of H2AX, a histone variant whose phosphorylation is a critical event in the signaling of DNA repair proteins, would lead to a GCR. We reasoned that increased spontaneous breaks in H2AX deficient cells might recombine with an engineered break in the F5 cells. Therefore, F5 cells were infected with an H2AX shRNA lentivirus, selected with puromycin for 48 hours, and assayed for inhibition of H2AX mRNA and protein (Supplemental Fig. 3). These cells were then transfected with an I-SceI expression vector, and single cell GCVR clones were identified by limiting dilution. 33 pure clones were isolated and characterized. Thirteen were false positive, and the remainder were interstitial deletions; no GCR were identified (Supplemental Table 1).

### A single engineered DNA DSB leads to GCR, including inversions and balanced translocations, in H2ax −/− cells

To evaluate that possibility that complete absence, as opposed to diminished amounts of H2ax protein, would allow frequent GCR, we transfected spontaneously immortalized murine H2ax −/− fibroblasts with the EF1aTKHyg vector, and isolated pure single cell clones. A clone that had integrated a single copy of the EF1aTKHyg vector and was verified to be GCV sensitive, designated H2ax8, was selected for further study. We used inverse PCR to clone the integration site, which demonstrated that the construct had been integrated on chromosome 11, nuc 105615 495 (NCBI36 assembly). This cell line was transfected with the pCBASce I-SceI expression vector, and 49 GCVR clones were obtained.

Of the 49 clones, 8 were false positive (no cleavage at the I-SceI site) (Table 2). Of the 41 remaining clones, 33 had insertions of vector sequence at the I-SceI site, and 3 clones had small interstitial deletions. Of the 33 clones that had captured vector sequences, 4 clones were represented twice, and one clone was represented 4 times. Thus, there were 26 independent clones that had become GCV resistant due to the repair of the I-SceI break via a “vector capture” event. Of the 3 clones with small interstitial deletions, two were identical. Therefore, a total of 28 independent clones were GCV resistant due to either a vector capture event or an interstitial deletion. However, the 5 remaining clones all had unique clonal, stable GCR, resulting in a frequency of 5/33 (15%) independent events. The characterization of these 5 clones is described in detail below.

**Figure 2:**
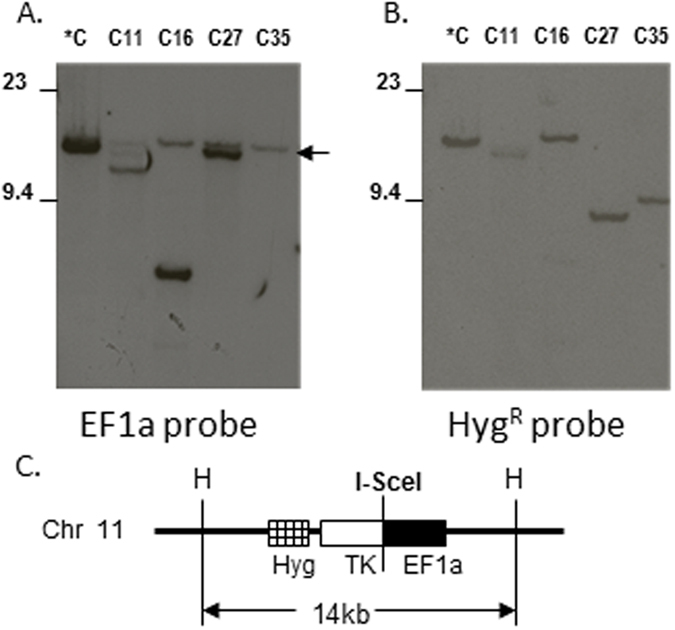
EF1a and HygroR fragments become separated after I-SceI transfection. Southern blot of the parental H2ax8 (*C) and subclones (C11, 16, 27, and 35), digested with HindIII, and hybridized to either an **(A)** EF1a probe or a **(B)** HygR probe. Arrow indicates cross-hybridization to the endogenous murine Ef1a gene. (**C**) Diagram of EF1aTKHyg vector integrated into chromosome 11. “H” indicated Hind III sites on mouse chromosome 11. Since the EF1aTKHyg vector lacks Hind III sites, the EF1a and HygR probes hybridize to the same Hind III fragment. Note that the hybridizing Hind III fragments are different in size for the 4 subclones, demonstrating separation of the EF1a and HygR fragments.

Figure 2 shows Southern blot hybridization of 4 of these 5 clones, compared to the parental clone. All 4 clones show the appearance of novel bands that are different in size than the wild-type size shown in the control lane. In addition, the size of the fragments that hybridized to the EF1a probe were different than the size of the fragments hybridizing to the Hygro probe, indicating that the Ef1a and Hygro sequences had become separated, and resided on different Hind III fragments.

**Figure 3:**
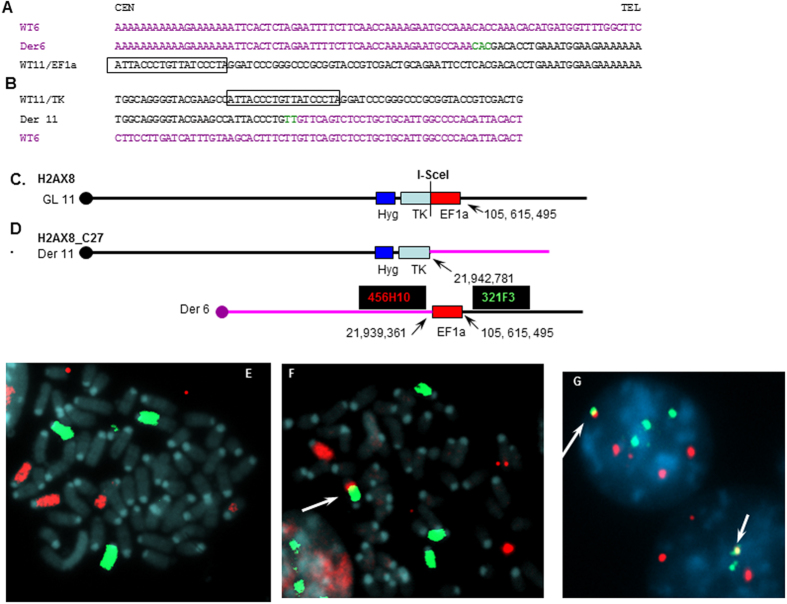
Clone C27 contains a balanced translocation. (**A**) Sequence of the wild-type (WT) chromosome 6, derivative 6 and EF1aTKHyg construct integrated on chromosome 11. Centromere and telomere are indicated; I-SceI site is boxed. Chromosome 11 sequences are in black; chromosome 6 sequences are in purple. Microhomologous nucleotides are in green. (**B)** Sequence of the integrated EF1aTKHyg construct, der(11), and WT chromosome 6. (**C**) Parental H2ax8 clone, with EF1aTKHyg integrated on chromosome 11. EF1a, TK, and HygR regions are indicated. (**D)** Subclone H2ax8_C27 with balanced translocation to chromosome 6 (pink). Derivative (der) 11 and 6 chromosomes and fusion points are indicated. (**E–F)**Chromosome 11 (green) and 6 (red) painting of H2ax8 (**E**) or H2ax8_C27 **(F)**. Arrow indicates der 11 fusion. (**G**) Der 6 identified by hybridization of H2ax8_C27 to 456H10 (red signal) and 321F3 (green signal) BAC clones; yellow fusions are indicated with arrows.

**Figure 4:**
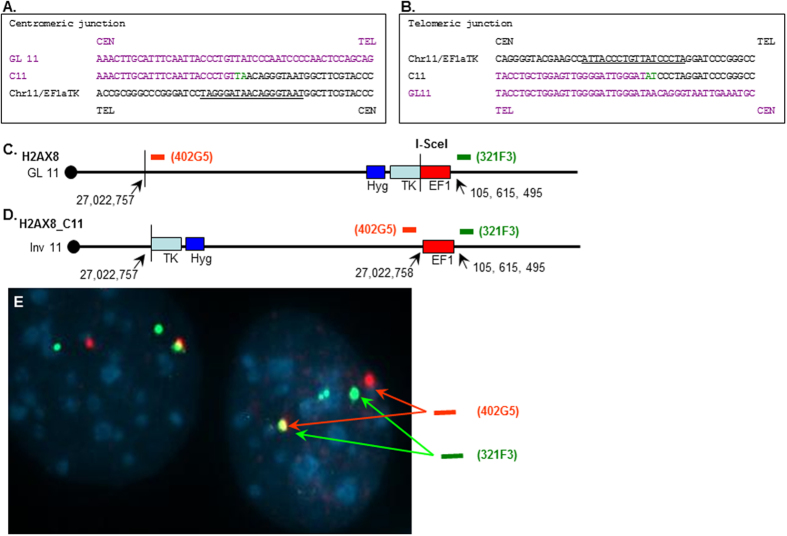
Generation of a 78 MB inversion. (**A)** Nucleotide sequence of the centromeric junction (fusion of TK sequences from clone C11 to germline chromosome 11). The I-SceI site is underlined, and 2 bp of microhomology are shown in green. The centromeric germline chromosome 11 seqeunces are shown in purple; the telomeric, EF1aTKHyg sequences are in black. Centromeric (CEN) and telomeric (TEL) orientation for the germline chromosome regions are indicated. (**B**) Nucleotide sequence of the telomeric junction (fusion of EF1a sequences to germline chromosome 11). (**C)** Integration of the EF1αTkHyg vector on chromosome 11. Positions of BAC clones 402G5 and 321F3 is indicated. (**D)** Inversion of H2ax8_C11 caused by break at nuc. 27,022,757. (**E)** Hybridization of H2ax8_C11 to 402G5 (red signal) and 321F3 (green signal) Note yellow signal due to overlap of probes.

Analysis of the H2ax_C27 clone is depicted in Fig. 3. Inverse PCR demonstrated that EF1a sequences from the integrated EF1aTKHyg construct had become joined to chromosome 6:21,939,362, and that TK sequences from the integrated construct joined to chromosome 6:21,942,781, suggesting that this rearrangement resulted from a balanced translocation, with deletion of 3,418 nucleotides of intervening chromosome 6 sequence (Figs 3A and 4B). Micro-homology was evident, with CAC and TT nucleotides on the derivative (der) 6 and 11 chromosomes respectively. Given that I-*Sce*I cleavage has been reported at off-target sites that have 1-5 bp mismatches for the 18 bp I-*Sce*I recognition sequence^27^, we inspected the sequence at the chromosome 6 break sites; no similarity to the I-*Sce*I recognition sequence was identified, even allowing for 6 mismatches. To verify that this rearrangement was indeed due to a chromosomal translocation, and not insertion of sequences derived from chromosome 6, we used chromosome painting and FISH (Fig. 3C–G). Figure 3F shows the derivative chromosome 11 fused to chromosome 6 sequences, and Fig. 3G shows juxtaposition of BAC clones from chromosome 6 and 11 on the der(6) chromosome.

Analysis of the H2ax_11 clone indicated that this rearrangement was due to a chromosomal inversion of 78 Mb (Fig. 4). The rearranged fragments shown in the Southern blot (Fig. 3) were cloned by inverse PCR, and the nucleotide sequence (Fig. 4A and B) suggested that the rearrangement was due to a large, 78 Mb inversion, depicted schematically in Fig. 4C and D. Microhomology (2 bp) was again evident at the breakpoint junctions. Similar to the breakpoints in Fig. 3, there were no similarities to the known I-*Sce*I recognition sequence. We used FISH to verify that this rearrangement was due to a chromosomal inversion (Fig. 4E). Similarly, we identified and characterized 3 additional clones that had undergone translocations or inversions (Supp Fig. 4–6).

### Correction of H2Ax deficiency protects cells from GCR

To determine if correction of the H2ax deficiency would protect cells from GCR, we transfected H2ax8 cells with an H2ax expression vector (pCMV3.1-H2ax-Puro), and selected single cell clones with puromycin. Treatment of two of these clones (Rh8-4 and Rh8-9) with the DNA-damaging agent staurosporine led to the gamma-phosphorylation of H2ax (Supp Figure 7), indicating that the H2ax deficiency seen in the H2ax8 cells had been functionally corrected. To determine whether the I-*Sce*I expression vector caused global DNA DSB, due to off-target effects, we transfected H2ax8 cells and the repaired Rh8-4 clone with an I-*Sce*I expression vector and assayed for H2ax phosphorylation. We saw no evidence of H2ax phosphorylation in either the H2ax cells or the Rh8-4 clone (Supp Figure 8). We then transfected the Rh8-4 clone (which had a functional H2ax protein) with the pCBASce expression vector, and selected GCV resistant clones.

36 clones were recovered and expanded. Of these, 25 had insertions of vector sequences at the I-SceI cleavage site (“vector capture”), three had undergone interstitial deletions, two had small TSIs^26^ and one had a large, 50 kb insertion (Supplemental Table 2). Of the two TSIs, one was derived from a LINE element, and one from a coding exon, similar to our previous findings^26^. The third clone had an insertion of almost 51 kb; the centromeric breakpoint was within an extended polypyrimidine repeat region of 103 bp, and the telomeric breakpoint was within a coding exon of the Ccdc137 gene. However, as opposed to the parental H2ax deficient cells, no chromosomal translocations or megabase inversions were recovered from the Rh8-4 cells, which had corrected the H2ax deficiency. To investigate whether H2ax deficiency influenced the cleavage efficiency of the I-*Sce*I enzyme, we transfected cells with the pCEP4-ISceI expression vector and assessed cleavage efficiency by PCR amplification across the I-*Sce*I site (Supplementary Figure 9). We did not detect a difference in cleavage efficiency between the H2ax8 and Rh8-4 cell lines; however, it should be noted that the cleavage efficiency may be below the limit of detection of the assay employed, and we therefore cannot exclude the possibility that absence of H2ax affected the I-*Sce*I cleavage efficiency

## Discussion

GCRs have been associated with malignant transformation and tumor progression. However, the mechanism(s) by which GCRs are caused, although being an active area of investigation, remains elusive. In prior studies we generated a single DNA DSB using I-SceI as described in the experiments above, and reasoned that a mis-repair event, that joined the single, experimentally engineered DNA DSB with a spontaneous DNA DSB might create a GCR. However, in our previous studies^21^,^22^,^26^, we did not detect any clonal, stable GCR produced by a single engineered DNA DSB. Subsequently, we used etoposide or bleomycin to generate random DNA DSBs, in conjunction with an I-SceI expression vector to produce a specific DNA DSB. However, despite the introduction of a second DNA DSB with bleomycin or etoposide, we identified no GCRs using this approach^21^,^22^.

Recently, two groups used I-SceI induced DNA DSB in combination with High -throughput genome wide translocation sequencing (HTGTS) or Translocation Capture Sequencing (TC-Seq) to detect DNA fusions in B lymphocytes^27^,^28^. In contrast to our results^21^,^22^,^26^. these investigators identified thousands of DNA fusions, many derived from distant chromosomal regions, consistent with chromosomal translocations. However, it should be noted that the experimental approaches used in those studies did not allow the recovery of clonal populations of cells, thus they were unable to determine whether the DNA fusions they recovered were stable rearrangements which could be passed on to daughter cells, or whether they represented balanced or unbalanced chromosomal rearrangements.

Because we were unable to generate stable, balanced chromosomal rearrangements with a single engineered DNA DSB in several cell lines, we turned our focus to H2AX deficient cells. H2AX is a histone variant representing approximately 10% of total histone H2A^29^. When DNA is damaged, H2AX variant histones, located at the site of the DNA DSB, become phosphorylated at serine 139. The phosphorylated H2AX, designated γ-H2AX, is not typically present in nucleosomes^29^,^30^,^31^ and is often used as a biomarker for DNA damage^32^. Previously, it has been determined that H2ax deficient B lymphocytes are impaired in their ability to repair DNA DSB activated by class switch recombination (CSR), and that a significant number of these CSR induced breaks are mis-repaired, leading to chromosomal translocations involving the Igh locus. Some of these translocations are oncogenic, resulting in the *in vivo* expansion of clones with oncogenic translocations^33^,^34^. Thus, we predicted that increased spontaneous DNA DSB events, which are known to be associated with H2AX deficiency, would allow the H2AX deficient cells to develop stable GCRs, in which an engineered DNA DSB induced by the I-*Sce*I enzyme was joined in a rare mis-repair event to a spontaneous DNA DSB present in the H2AX deficient cells. In the present study, we did not detect any balanced GCRs in H2AX knockdown cells. However, in cells with a complete ablation of H2ax, we found that aberrant repair of a single engineered DNA DSB produced clonal, stable GCRs; approximately 15% of single cell clones with mis-repair events leading to loss of TK expression showed a GCR. This predisposition was corrected by enforced expression of H2ax protein mediated via introduction of an H2ax expression vector. To determine whether or not stable GCRs could be produced by inhibition of “classical” NHEJ, we disrupted the NHEJ repair pathway through RNAi-mediated inhibition of KU80 and LIG4. KU80 not only detects DNA DSBs, but also recruits other core NHEJ factors to the site of a DNA DSB, while LIG4 is required to seal the DNA DSB in the final step of NHEJ-mediated repair of a DNA DSB^35^,^36^,^37^. Previous reports demonstrated that mouse embryo fibroblasts (MEFs) derived from mice that were devoid of both Lig4 and Tp53 (Lig4−/−Tp53−/−) showed a marked increase in non-reciprocal chromosomal translocations^38^, and Ku80−/−Tp53−/− mice developed pro-B cell lymphomas with chromosomal translocations^39^. The generation of GCRs in these settings is thought to be due to inhibition of “classical” NHEJ, and use of an “alternative” NHEJ^40^,^41^. However, RNAi-mediated inhibition of either Lig4 or Ku80, in conjunction with an I-SceI engineered DNA DSB, did not lead to GCR in the F5 monocytic cell line. The lack of GCRs in this system may be due to a number of possibilities. It may be that complete ablation of classical NHEJ is required, or the difference may be due to cell type (MEF or B-cell vs monocyte), or a selective advantage for rare Igh-Myc translocations that expand *in vivo*.

The mechanism by which H2ax deficiency promotes mis-repair of DNA DSB, leading to balanced GCRs, remains unclear. γ-H2ax serves as docking sites for several damage response proteins such as INO80 and SWR complexes, through association with Nhp10 and Arp4 subunits^42^,^43^. INO80 and SWR are ATP-dependent chromatin remodeling complexes that evict or slide nucleosomes to alter the structure of chromatin, forming nucleosome free regions (NFRs)^44^,^45^, allowing NHEJ factors access for repair of DNA DSBs. Several lines of evidence have demonstrated an interaction between INO80 complex and H2AX^46^. In the absence of H2ax, the presence of canonical nucleosomes hinders access of DNA damage repair factors to the DNA DSB. It is anticipated that the I-SceI restriction site is nucleosome free since it can be cleaved by I-Sce I protein, and it seems likely that delayed access to a DNA DSB leads to persistent DNA DSB, allowing a spontaneous DNA DSB to become juxtaposed with the DNA DSB generated by I-SceI.

In summary, we found that induction of a single engineered DNA DSB led to generation of clonal, stable GCRs, in the context of H2ax null cells. Interestingly, we found no evidence of GCRs in cells with diminished but still detectable levels of H2ax, suggesting that complete ablation of H2ax might be required to produce an environment that was permissive for generation of GCRs. The generation of these GCRs was prevented by re-expression of H2ax in the H2ax null cells. We predict that a lack of H2ax suppresses normal DNA DSB repair, perhaps by abrogating the normal interaction of H2ax with chromatin remodeling complexes such as the INO80 complex. This work supports the assertion that H2ax plays a critical role in maintaining the integrity of genetic information.

## Materials and Methods

### Cell lines, plasmids, and BAC clones

F5 cells were generated as previously described [19], and maintained in RPMI 1640 medium (Invitrogen) supplemented with 10% fetal bovine serum, 2 mm l-glutamine (Invitrogen), 1% penicillin and streptomycin (Invitrogen) and 300 ug/ml of G418 (Invitrogen). HEK 293 T cells were obtained from American Type Culture Collection and grown in DMEM medium (Invitrogen) supplemented with 10% fetal bovine serum, 2 mm l-glutamine (Invitrogen), 1% penicillin and streptomycin (Invitrogen). H2ax deficient fibroblasts were a kind gift from Dr. Andre Nussenzweig, and were generated as previously described^47^ and grown in DMEM media supplemented as above. The pEF1aTK plasmid and I*Sce*I expression vectors (pCBASce and pCEP4-ISceI) were previously described^19^. The pEF1aTKHyg plasmid was generated from the EF1aTK vector by digestion of the EF1aTK vector with SspI and NotI, isolating a 2.7 kb fragment containing the EF1a and TK cassette, and ligating the fragment to the pcDNA3.1/Hygro( + ) vector (Invitrogen) backbone. The H2ax expression vector (pRC-H2ax) was based on the pRNA-CMV3.1/Puro Vector (Gen Script) backbone. The H2ax insert (599 bp) was amplified from NIH3T3 DNA using primers BamH-H2AXF1 (5′ATAGGATCCGTGGTCTCTCAGCGTTGTTCGC-3′) and HindIII-H2AXR1 (5′-GGTAAGCTTAAGCCGCCGCAGCCCGAAGTGG-3′) to generated a PCR product containing BamHI and HindIII sites. Both the pRNA-C V3.1/ Puro vector and the H2ax PCR product were digested with BamHI and HindIII, purified, and ligated to create the pRC-H2ax expression vector. The vector was sequenced to verify the H2ax insert and orientation. Murine and human BAC clones were obtained from BACPAC resources, and BAC DNA was isolated using Qiagen reagents and protocols.

### Transfections and lentiviral transductions

For knockdown experiments using the F5 cells, pre-validated siRNA oligonucleotides (Qiagen) [5′-AAG CAT AAC TAT GAG TGT TTA-3′ (Hs_XRCC5_6 siRNA) or 5′-CAC GAC TAG AAC CTT AGG CAG-3′ (Hs_H2AFX_3 siRNA)] or a scrambled Negative Control (Qiagen; UUCUCCGAACGUGUCACGUdTdT) siRNA were co-transfected with the pCBASce vector. 4.5 ul of a 10 mM siRNA and 2 ug of pCBASce were mixed and transfected into 1 × 10^6^ F5 cells in 100 ul of Nucleofector solution C (Amaxa) using the W-01 Nucleofector program. The transfections were performed in duplicate or triplicate, in order to obtain sufficient cells, and then pooled. Forty-eight to seventy two hours later, cells were harvested and total RNA and protein were isolated. For knockdown of LIG4, a lentiviral vector expressing LIG4 shRNA (TRCN000004005) or the MISSION^®^ pLKO.1-puro Non-Mammalian shRNA control vector (Sigma) were transfected into HEK 293 T cells in a cocktail containing 20 ug of lentiviral shRNA transfer vector, 7.5 ug of the ∆8 vector, and 2ug of VSV-G. Lentiviral particles were collected after 48 hours, concentrated, and used to infect F5 cells. After incubation for 4 days, puromycin was added to a concentration of 4 ug/ml; following 7 days selection, total RNA and protein were harvested to determine knockdown efficency. 1 × 10^6^ cells shRNA transduced cells were then transfected with 2 ug of the pCBASce vector using Nucleofector reagents as described above. Four days after transfection of the I-SceI expression vector (to allow for the TK protein to decay), cells were plated in 96-well plates at a density of 0.7, 7, 70 and 700 cells/well, and selected with 50 uM GCV. The cells were allowed to expand for 2–3 weeks; GCV resistant clones were selected and genomic DNA was harvested.

To generate H2ax deficient cells containing the EF1aTKHyg vector, EF1aTKHyg was linearized with SspI, and 1 × 10^6^ H2ax −/− fibroblasts were transfected with 2 ug of vector in a volume of 100ul using Nucleofector solution V (AmaxaVCA-103) and the T-30 program. Cells were then selected in 300 ug/ml hygromycin (Invitrogen), and individual hygromycin resistant clones were isolated and expanded. To correct the H2ax deficiency in the H2ax8 clone, H2ax8 cells were transfected with the pRC-H2ax expression vector and selected with puromycin (5 ug/ml; Clontech). To induce a DNA DSB, H2ax8 cells that were approximately 80% confluent were transfected with the pCBASce vector using Lipofectamine 2000 (Invitrogen) reagent and a modified protocol, in order to avoid the “bystander effect” previously reported with GCV^48^. The day following transfection, cells were transferred to a T75 flask and allowed to recover and expand for 48 hrs. The following day (72 hrs post transfection), cells from the T75 flask were split 1:10 and plated in 10 cm dishes. GCV was added after the cells had attached (approximately 4 hrs), to a concentration of 20 uM. These experiments were repeated four times in order to obtain sufficient numbers of clones to analyze, and the results of five experiments are presented as pooled results.

### Quantitative RT-PCR

Total RNA was isolated using Trizol (Invitrogen) reagents and protocols. Genomic DNA contamination was eliminated with DNase (Ambion). One ug of total RNA and 50 ng of random hexamers were used to synthesize first strand cDNA in a volume of 20 uL, using Superscript Reverse Transcriptase (Invitrogen). Quantitative PCR was performed in 7500 real time PCR system (Applied Biosystems) using Taqman Fast universal PCR master mix (part No. 4352042, (Applied Biosystems). The reaction was 95 °C for 20 seconds, followed by 40 cycles at 95 °C for 3 seconds and 60 °C for 30 seconds. The primer/probe for KU80, DNA LIG 4 and H2AX were Hs0021707_m1, Hs00172455_m1 and Hs00266783_s1 (Applied Biosystems), respectively. Expression was normalized to the 18 S ribosomal RNA (Applied Biosystems #4308329).

### Western blotting

Cells were washed with PBS, and then re-suspended in RIPA lysis buffer (SC-24948) (Santa Cruz Biotechnology, Inc) including 1:1000 protease inhibitor cocktail, PMSF and Sodium orthovanadate (SC-24948). The mixture was incubated on ice for 30 minutes and cells were further disrupted using a 21-gauge needle. For H2AX extraction, the pellet was lysed in 0.2 N HCl overnight at 4 °C and neutralized with 2 M NaOH. The lysed cells were boiled for 5 minutes after adding Tris-glycine SDS Sample Buffer and centrifuged at 12,000 x g for 5 minutes. The proteins were resolved in 14% Tris-glycine gel (Invitrogen) and transferred to nitrocellulose membranes. The membranes were incubated with a 1:1000 dilution of goat polyclonal antibody to Ku80 (Ku-86 (M-20), sc-1485 Santa Cruz Biotechnology, Inc), 1:500 dilution of rabbit polyclonal antibody to LIG4 (ab26039, abcam), 1:2000 dilution of rabbit anti-Histone H2A.X polyclonal antibody (Cat. 07-627, Millipore), antiphospho-histone H2AX (ser139; clone JBW301, Millipore), anti-histone H3 (mABcam 10799; Abcam) or 1: 1000 dilution of rabbit Tubulin antibody (Cell Signaling), followed by 1:5000 horseradish peroxidase-conjugated rabbit anti-goat IgG, goat anti-rabbit IgG or rabbit anti-mouse IgG (Themo Scientific). Proteins were detected by chemiluminescence with Enhanced Chemiluminescence Reagent (Themo Scientific) and Kodak X-AR film.

### Fluorescence *in situ* hybridization (FISH)

Metaphase chromosome spreads were generated from the H2AX8 and derivative cell lines using conventional cytogenetic techniques and subsequently pretreated and denatured as described elsewhere (Roschke *et al*, 2002). BAC DNA was labeled by nick translation with digoxigenin-11-dUTP or biotin-16-(Roche Applied Science), precipitated in ethanol with 50 × excess of human Cot-1 DNA (Invitrogen), and resuspended to a final concentration of 50 ng/μl in Hybrizol solution (MP Biomedicals). Whole chromosome painting probes for were obtained from Applied Spectral Imaging. After denaturing at 80 °C for 10 min and preannealing at 37 °C for 60 min, 10 μl of probe mixtures were applied under 22 mm × 22 mm coverslips. Slides were incubated in a moist chamber overnight at 37 °C. After detection with anti-digoxigenin antibodies labeled with rhodamine (Roche Applied Science) or avidin conjugated with FITC (Roche Applied Science) they were mounted in antifade solution (Vector Laboratories) containing DAPI. A Leica microscope equipped with DAPI, FITC and rhodamine filters (Chroma Technology) and a Sensys CCD camera (Photometrics) connected with Q-FISH software (Leica Microsystems Imaging Solutions) were used for image acquisitions.

## Additional Information

**How to cite this article**: Qiu, Z. *et al*. Generation of Gross Chromosomal Rearrangements by a Single Engineered DNA Double Strand Break. *Sci. Rep.* **7**, 43156; doi: 10.1038/srep43156 (2017).

**Publisher's note:** Springer Nature remains neutral with regard to jurisdictional claims in published maps and institutional affiliations.

## Supplementary Material

Supplemental Information

## Figures and Tables

**Table 1:** DNA rearrangements produced by concurrent I-*Sce*I cleavage and NHEJ inhibition.

NHEJ Protein	Number of clones	False positive	Interstitial deletion	Insertion	Local Inversion	Megabase deletion
***Ku80***	189	124 (66)	63 (33)	1 (<1)	1 (<1)	0
***LigIV***	130	36 (28)	92 (71)	0	1 (<1)	1 (<1)
**Control**	49	31 (63)	18 (37)	0	0	0

Percentages indicated in parentheses.

**Table 2:** GCV^R^ clones recovered after transfection of H2ax8 cells with an I-Sce-I expression vector.

Viable clones	WT	Interstitial Deletion	Vector Capture	Inversion	Translocation
49	8 (16)	3 (6)	33 (67)	3 (6)	2 (4)

WT—wild-type; no cleavage at I-SceI site.

Percentages are indicated in parentheses.
