# Supplementary material for: Generation of Gross Chromosomal Rearrangements by a Single Engineered DNA Double Strand Break
Source: Sci Rep. 2017 Feb 22;7:43156. doi: 10.1038/srep43156 (PMC5320478; doi:10.1038/srep43156)
Supplement: Supplemental Information [file srep43156-s1.pdf]

Supplementary Information

Generation of Gross Chromosomal Rearrangements by a Single

Engineered DNA Double Strand Break

Zhijun Qiu1, Zhenhua Zhang1, Anna Roschke1, Tamas Varga1, and Peter D. Aplan1*

[1]Genetics Branch National Cancer Institute, National Institutes of Health, Bethesda, MD 20892, USA

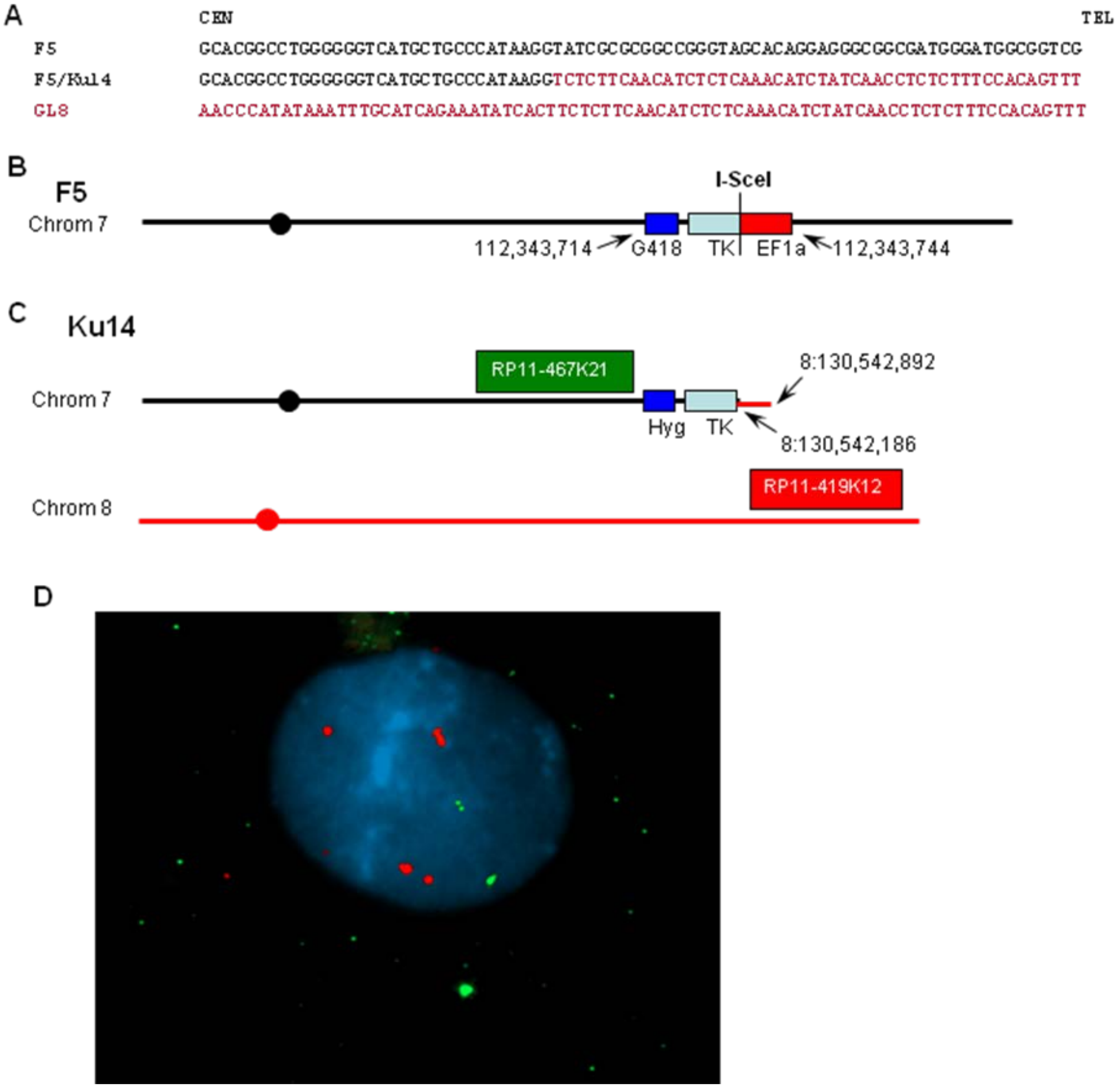

Supplemental figure 1. No evidence for chromosomal translocaiton of clone Ku14. A) Nucleotide sequence of the EF1aTK vector integrated into chromosome 7 (parental clone F5), breakpoint junction of the Ku14 clones identified by inverse PCR, germline chromosome 8 sequences from the breakpoint region. TK sequences are in black, germline chromosome 8 sequences are in red. **B)** Diagram of the parental clone F5; integration sites and orientation of the EF1aTK vector are indicated. Coordinates are from NCBI build 34. **C)** Diagram of the Ku14 clone. Location of the Bac clones are indicated. Fusion to chromosome 8 sequences are indicated. **D)** Hybridization of the Ku14 clone with chromosome 7 (green) and chromosome 8 (red) probes shows no co-localization.

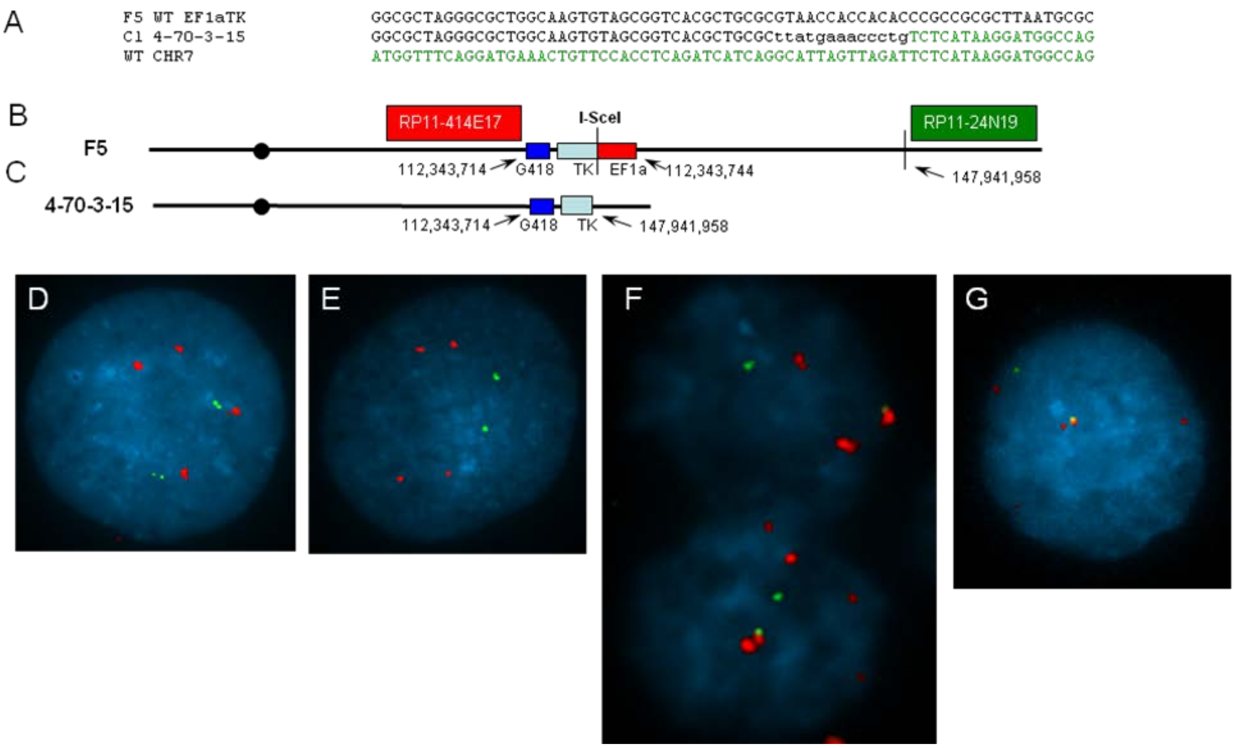

Supplemental Figure 2. 35 Mb interstitial deletion in clone 4-70-3-15. A) nucleotide sequence of the integrated EF1aTK vector, clone 4-70-3-15, and germline (GL) chromosome 7, distal to the EF1aTK integration site. **B)** Schematic representation of EF1aTK vector integrated on chromosome 7. Nucleotide positions refer to NCBI34 assembly. **C)** Representation of interstitial deletion in clone 4-70-3-15. **D-E)** Hybridization of BAC clones RP11-414E17 (red) and RP11-24N19 (green) to parental F5 cells. Note the wide separation of signals. **F-G)** Hybridization of BAC clones RP11-414E17 (red) and RP11-24N19 (green) to clone 4-70-3-15. Note the yellow/orange overlap of signals in all 3 interphase cells

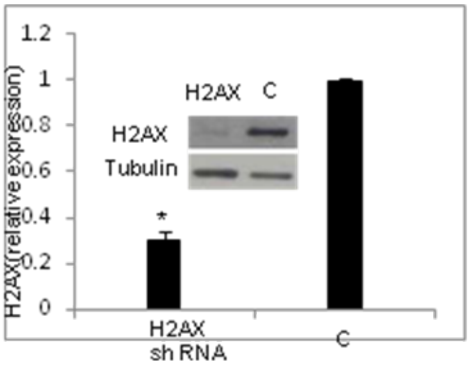

Supplemental Figure 3. Inhibition of H2AX by shRNA. F5 cells were infected with a lentiviral shRNA, and H2AX mRNA was assessed by RQ-PCR, and Western blotting (inset). Tubulin was used as a loading control for the Western blot. *, $p<0.01$. Blot cropped to improve clarity and conciseness.

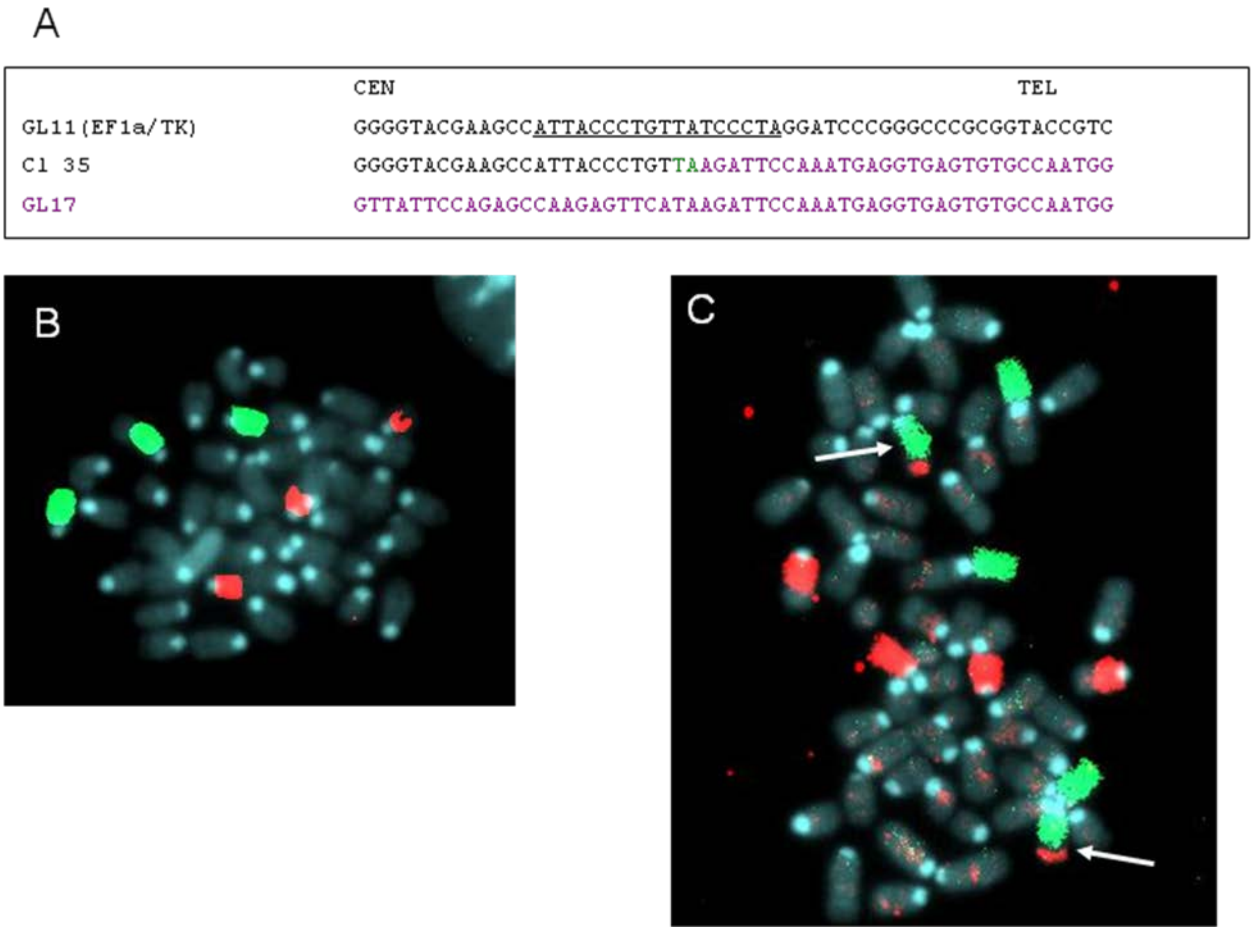

Supplemental Figure 4. Clone 35 results from an unbalanced translocation. A) Nucleotide sequence of the EF1aTKhyg construct integrated on chromosome 11, germline chromosome 17, and the fusion at nucleotide 72,958,178 (NCBI Build 36) . Microhomology (2bp) is indicated in green. Centromeric and telomeric orientation are indicated. **B)** Chromosomal painting of H2ax8 parental clone with chromosome 11 (green) and chromosome 17 (red). **C)** Chromosome painting of H2ax8_C35; arrows indicate 2 copies of the der(11) chromosome.

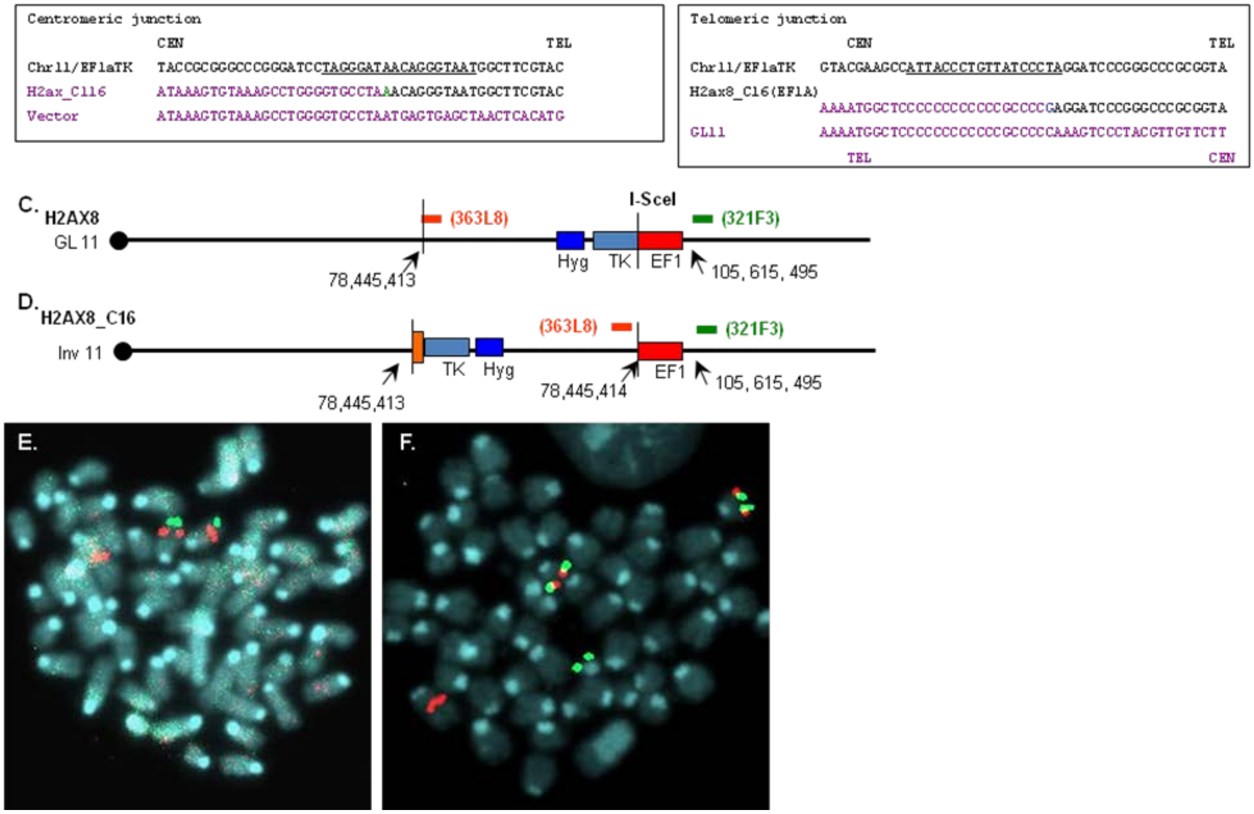

Supplemental Figure 5. Generation of 27 MB inversion in H2ax8_C16 clone. A. Nucleotide sequence of the centromeric junction (fusion of TK sequences to I-SceI expression vector sequences). The I-SceI site is underlined, and 1 bp of microhomolgoy is shown in green. Centromeric (CEN) and telomeric (TEL) orientation for the germline chromosome regions are indicated. **B.** Nucleotide sequence of the telomeric junction (fusion of EF1a sequences to germline chromosome 11). The I-SceI site is underlined and 1 bp non-templated nucleotide is indicated in blue. 11). **C.** Integration of the EF1αTkHyg vector on chromosome 11. Positions of BAC clones 363L8 and 321F3 are indicated. **D.** Inversion of H2AX8_C11 caused by break at nuc. 78,445,414**. E.** Hybridization of H2AX8_C11 to 363L8 (red signal) and 321F3 (green signal) to parental H2ax8 cell line. Note that the red signal is centromeric to the green signal. **F.** Hybridization of the BAC clones to H2ax8_C16. Note yellow signal due to overlap of probes.

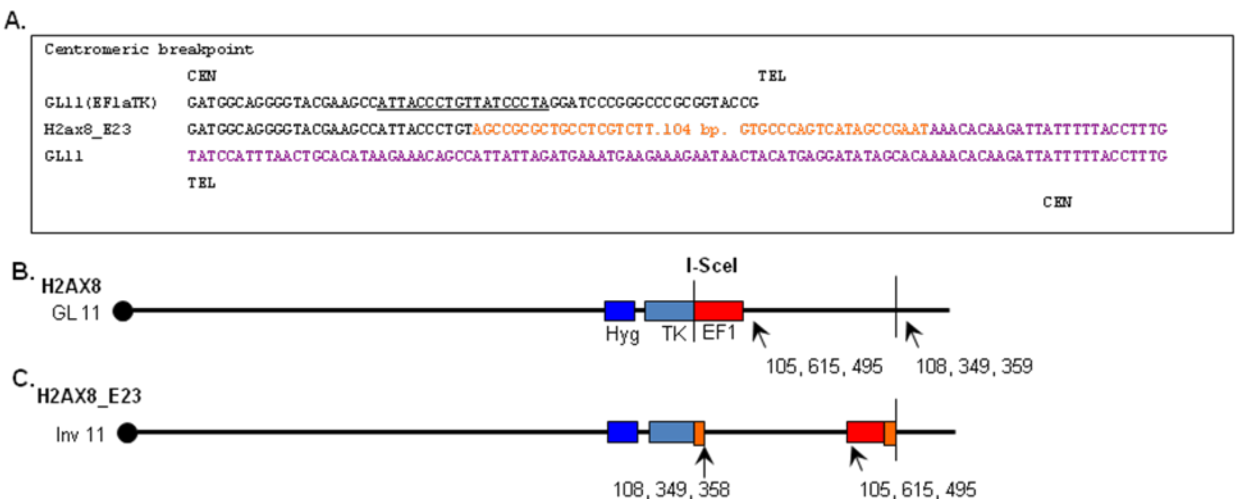

Supplemental Figure 6. Generation of 2.7 MB inversion in H2ax8_E23 clone. A. Nucleotide sequence of the centromeric junction due to fusion of TK sequences to chromosome 11 at nucleotide 108,349,358. 144 bp of sequence derived from the expression vector (indicated in orange) is interspersed between the TK sequences and chromosome 11 sequences. The I-SceI site is underlined and centromeric (CEN) and telomeric (TEL) orientation for the germline chromosome regions are indicated. **B.** Integration of the EF1αTkHyg vector on chromosome 11. **C.** Inversion of H2AX8_E23 caused by break at nuc. 108,349,358. Sequences derived from the I-SceI expression vector are indicated in orange.

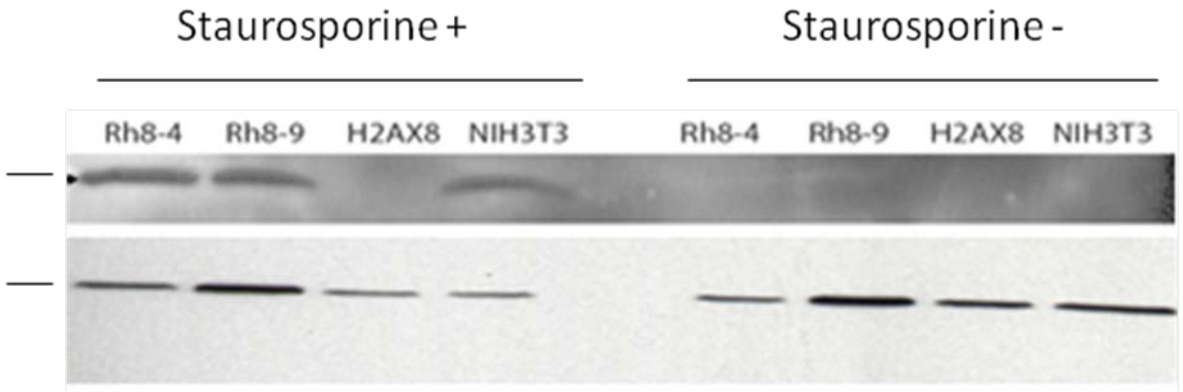

Supplemental Figure 7: Correction of H2ax deficiency. Clones transfected with the pCMV-H2ax-Puro vector (designated Rh8-4 and Rh8-9) were treated with 0.5 uM staurosporine for 6 hrs. Parental H2Ax8 and NIH3T3 cells were used as negative and positive controls respectively. Blots were probed with anti-phospho-H2AX (upper panel) or tubulin (lower panel) as a loading control. Blots cropped to improve clarity and conciseness.

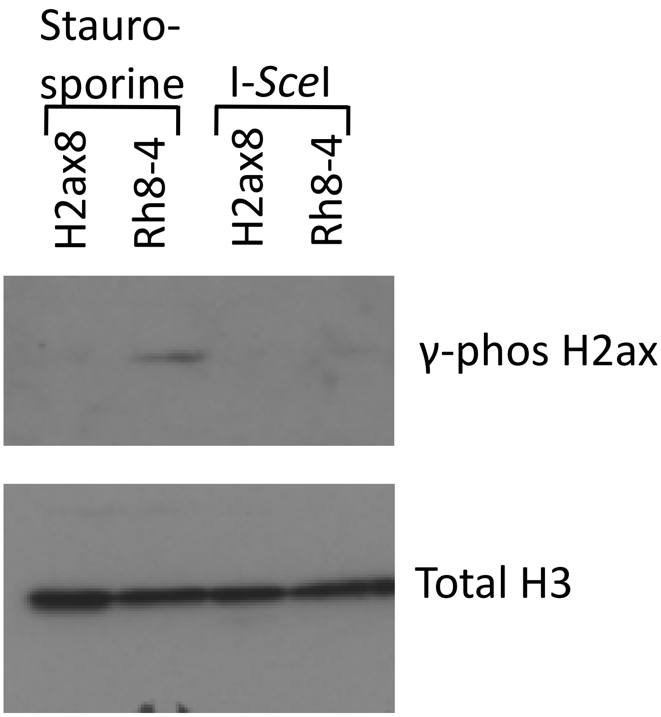

Supplemental Figure 8. Transfection with I-SceI does not induce γ-phosphorylated H2ax. H2ax8 and daughter clone Rh8-4 cell lines were transfected with the pCEP4-ISceI episomal plasmid and allowed to recover for 48 hours. Acid extracted lysates were assessed for γ-phosphorylated H2ax. Total histone H3 was used as a loading control. H2ax8 and Rh8-4 were treated with 0.5 uM staurosporine as a positive control. Blots cropped to improve clarity and conciseness.

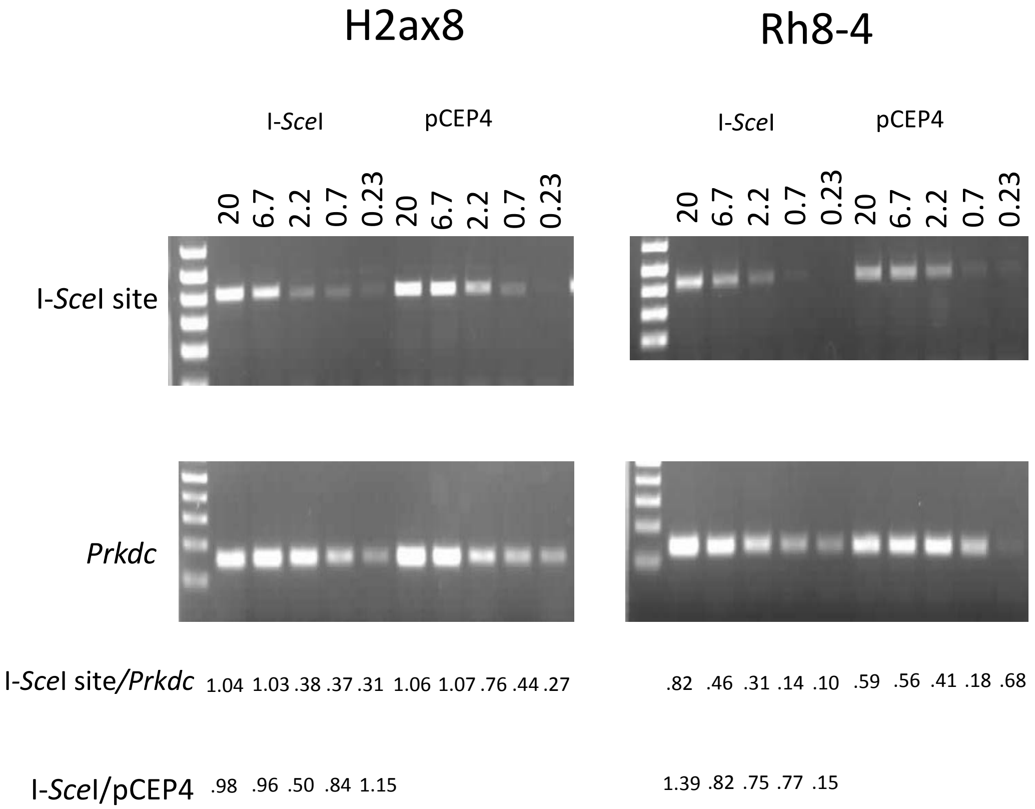

Supplemental Figure 9. Efficiency of I-*Sce*I cleavage. The H2ax8 and Rh8-4 cell lines were transfected with an I-*Sce*I expression vector or empty vector control (PCEP4). Following a 48 hour recovery period, integrity of the I-SceI site was assessed by PCR amplification of a dilution series of genomic DNA (20, 6.7, 2.2, 0.7, 0.23 ng) across the I-*Sce*I site using primers Tkseq (5'-AACGCCGTACGTCGGTTGCTATGG-3') and EFcheckF2 (5'-TTCATGTGACTCCACGGAGTACCG-3'), normalized to amplification of a genomic *Prkdc* fragment as an internal control (using primers 5'-GGAAGAGTTTTGAGCAGACAATG-3' and 5'-CATCACAAGTTATAACAGCTGGG-3') , and quantified using Image J software (Research Services Branch, National Institute of Mental Health, Bethesda, Maryland, USA). Ethidium bromide stained gel cropped to improve clarity and conciseness. The efficiency of cleavage was estimated by comparing the intensity of the fragment containing the I-*Sce*I site in cells transfected with the I-*Sce*I vector to the intensity of the fragment in cells transfected with the pCEP control vector. Although there was a trend toward increased cleavage of the I-*Sce*I site (reflected by decreased amplification of the fragment) in the Rh8-4 cells compared to the H2ax8 cells, this difference was not statistically significant (Student's t test, $p>0.05$)

Supplemental Table 1. DNA rearrangements produced by expression of I-SceI after H2AX knockdown

Number of clones	False positive	Interstitial deletion	GCR
33	13	20	0

Supplemental Table 2. Repair of DNA DSB in RH8-4 cells

Viable clones	WT	Interstitial deletion	Vector Capture	Small insertion	Large insertion
36	5	3	25	2	1

Supplemental Table 3. Insertions derived from distant regions of the genome

Clone #	Microhomology	Non-templated nucleotides	Inserted region	Notes on inserted region
9	1 bp,2 bp	0 bp, 0 bp	9:17,963,340-17,963,705	LINE element
56	0 bp, 0 bp	0 bp, 0 bp	10:79737732-79737948	Reep6 exon 5
**50**	**0 bp, 0 bp**	**2 bp, 0 bp**	**11:120,276,293-11:120,327,221**	**Extended (103 bp) polypyrimidine tract; Ccdc137 exon 3**
